# A Flexible Metamaterial Absorber via Loss Engineering for Large‐Area Ultra‐Broadband Infrared Extinction

**DOI:** 10.1002/advs.75513

**Published:** 2026-05-07

**Authors:** Zhe Wu, Zhongzhu Liang, Xiaoyan Shi, Fuming Yang, Enzhu Hou, Jihui Jiang, Xintong Wei, Siyu Guo, Bing Sun, Qingxin Tang, David R. Smith, Haiyang Xu

**Affiliations:** ^1^ State Key Laboratory of Integrated Optoelectronics and Key Laboratory of ultraviolet Light‐Emitting Materials and Technology of Ministry of Education College of Physics Northeast Normal University Changchun China; ^2^ Center for Metamaterials and Integrated Plasmonics Department of Electrical and Computer Engineering Duke University Durham North Carolina USA

**Keywords:** flexible photonics, infrared absorption, loss engineering, metamaterial absorber, stray light elimination

## Abstract

The advancement of compact and wearable optical systems is critically limited by the lack of high‐performance, integrable solutions for managing stray light, particularly in the mid‐infrared regime. Traditional approaches relying on bulky baffles or cryogenic cooling hinder miniaturization and flexibility. Herein, we report a large‐area, flexible metamaterial film based on a Ti/Al_2_O_3_/Fe_3_O_4_/Ti heterostructure that functions as an ultra‐broadband infrared extinction layer. By introducing a lossy Fe_3_O_4_ interlayer, our design achieves an exceptional average absorptivity of 97.1% across the 3–5 µm atmospheric window, with a total thickness below 1 µm. This “loss engineering” strategy effectively broadens the absorption bandwidth and smoothens the spectral response compared to conventional metal‐insulator‐metal(MIM) absorbers. The film is fabricated on a flexible polyimide substrate via scalable lithography, demonstrating remarkable mechanical robustness. More importantly, we integrate it into a practical optical system as a cylindrical baffle, where it suppresses stray light intensity to a mere 0.6% after three reflections, significantly enhancing imaging contrast. This work presents not merely a high‐performance absorber, but a scalable, flexible material platform that paves the way for next generation miniaturized and flexible optical devices.

## Graphical abstract

Designed and demonstrated a flexible metamaterial absorber via loss engineering for large‐area ultra‐broadband infrared extinction, providing a new method for miniaturization of absorbers and flexible optical devices.

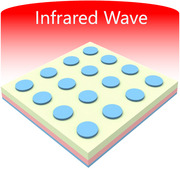

## 1 Introduction

The relentless pursuit of miniaturization and flexibility in modern optical systems, such as portable infrared spectrometers, wearable sensors, and compact imaging units, has created a pressing demand for components that are both highly functional and mechanically compliant [1, 2, 3]. A paramount challenge in these systems is the effective suppression of stray light—unwanted radiation that degrades image contrast, reduces signal‐to‐noise ratio, and compromises accuracy in applications ranging from infrared remote sensing to machine vision [4, 5, 6, 7, 8, 9]. Conventional stray light management strategies, including the use of complex multi‐stage baffles and black coatings, inherently contradict the goals of miniaturization and light‐weighting due to their significant volume and rigidity [10, 11, 12]. Furthermore, in infrared systems, the thermal emission from the components themselves necessitates additional cryogenic cooling, adding further complexity and cost [13, 14, 15, 16, 17, 18]. While alternative strategies like quantum dots have been explored, their performance often degrades under wide‐angle incidence [19, 20]. There is, therefore, an urgent need for a paradigm‐shifting solution: a thin, lightweight, and flexible material that can be seamlessly integrated into optical paths to provide broadband extinction of stray light.

Metamaterial absorbers, particularly those with MIMconfigurations, have emerged as a promising candidate for perfect light absorption due to their ability to tailor electromagnetic response through subwavelength structures [21, 22, 23]. However, their practical application in flexible optical systems faces two major hurdles: achieving ultra‐broadband, high‐performance absorption with a simple structure amenable to large area fabrication, and endowing the device with mechanical flexibility without sacrificing optical performance. Most reported MIM absorbers exhibit narrowband resonance or require complex multi‐resonator designs, which are difficult to scale up [24, 25, 26, 27, 28, 29]. Moreover, they are typically fabricated on rigid substrates, limiting their integration potential.

To overcome these limitations, we propose a material‐level innovation by introducing a lossy dielectric layer into a classic MIM structure. We demonstrate a large‐area, flexible metamaterial film based on a Ti/Al_2_O_3_/Fe_3_O_4_/Ti heterostructure. The incorporation of Fe_3_O_4_, a material with a significant imaginary part of the refractive index in the infrared regime, serves as a “loss engine” that effectively broadens the absorption bandwidth through enhanced dielectric loss. This design enables ultra‐broadband absorption with a simple, single‐sized circular pattern, making it compatible with large‐area lithography. The film, with a total thickness of less than 1 µm, is fabricated on a flexible polyimide substrate, demonstrating excellent mechanical properties. Crucially, we integrated the film into a practical stray light elimination scenario, and the results show that our flexible extinction tube can reduce stray light intensity to 0.6%, dramatically improving the contrast in infrared imaging. This work highlights the potential of loss‐engineered metamaterial films as a versatile material platform for revolutionizing stray light management in future flexible and compact optical systems.

## 2 Results and Discussion

### 2.1 Structural Design and Broadband Absorption of the Ti/Al_2_O_3_/Ti Metamaterial

**FIGURE 1:**
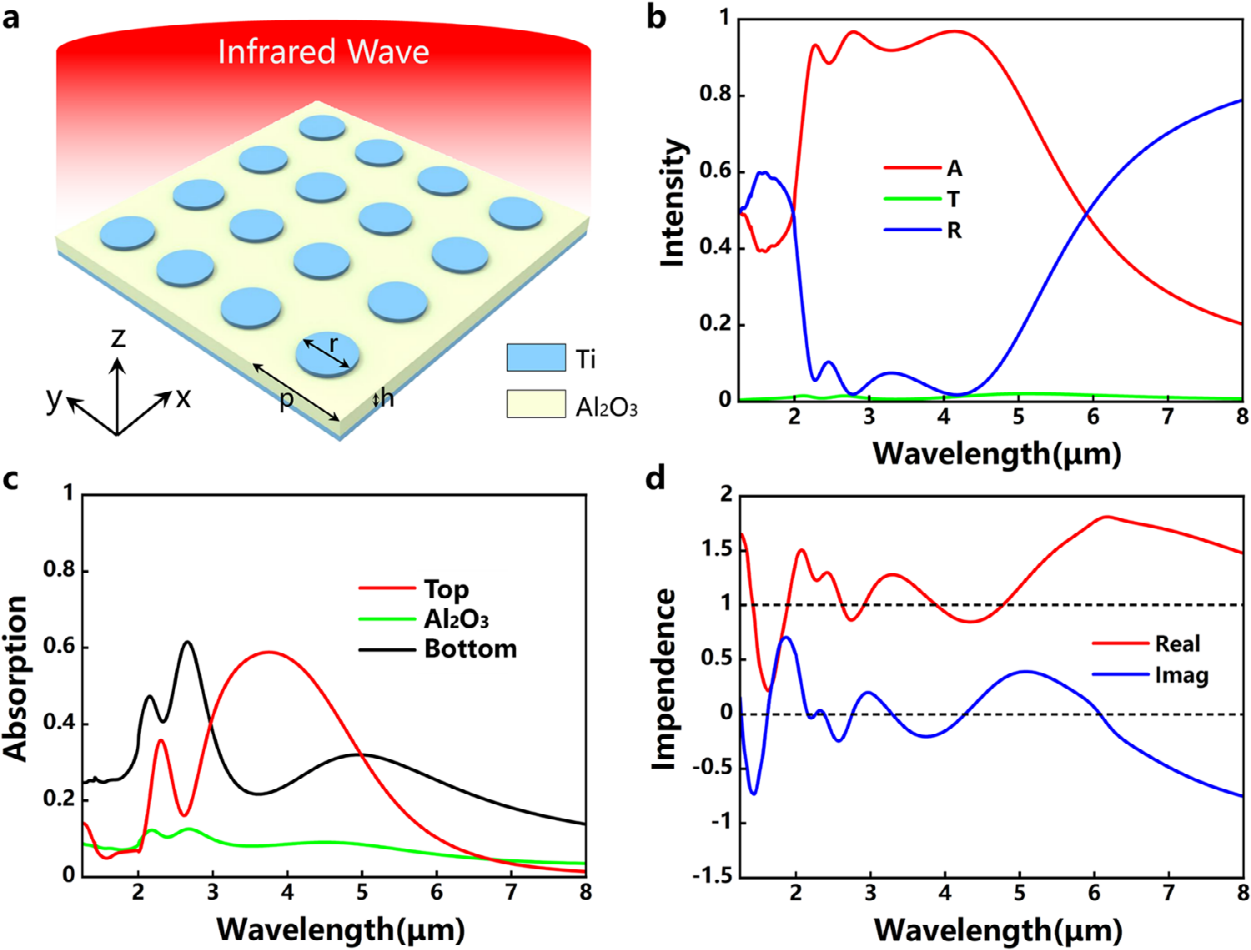
Characteristics of the Ti/Al_2_O_3_/Ti absorber. a) Structural diagram of the proposed Ti/Al_2_O_3_/Ti absorber with the periodic circular top resonators. Specifically, *r* = 1 µm, *p* = 2 µm and *h* = 400 nm. The thickness of the top Ti layer is 100 nm, and the bottom Ti layer is 100 nm, which is thicker than the penetration depth. b) Absorption, transmission and reflective spectrum of the proposed Ti/Al_2_O_3_/Ti absorber. c) The contribution of each component of the proposed Ti/Al_2_O_3_/Ti absorber to absorption. d) The equivalent impedance of the proposed Ti/Al_2_O_3_/Ti absorber.

Figure 1 delineates the fundamental absorption characteristics of the foundational Ti/Al_2_O_3_/Ti metamaterial structure. The schematic in Figure 1a presents the triple‐layer configuration with periodic circular Ti patches (radius *r* = 1 µm, period *p* = 2 µm). The simulated absorption spectrum in Figure 1b reveals three distinct resonance peaks at 2.26 µm (93.3%), 2.80 µm (96.7%), and 4.05 µm (96.9%), culminating in an average absorptivity of 93.1% across the 3–5 µm band, while the transmission is effectively suppressed to near zero by the optically thick bottom Ti layer. The broadband absorption of the absorber is obtained through the excitation of mixed resonant modes by the resonator array. That is to say, the incident light is coupled to the resonator array, causing the incident electric and magnetic fields to concentrate within the absorber structure, resulting in ohmic losses caused by surface currents. We calculated the absorption of incident light energy by each layer using equation [30]:

(1) $$Q\left(\omega \right)=\frac{1}{2}\times \omega \times \varepsilon^{\prime \prime }\times {\left|E(\omega )\right|}^{2}$$

here, *ω* is the angular frequency, *ε*′' is the imaginary part of the permittivity of the material, and *E*(*ω*) represents the electric field strength corresponding to the angular frequency *ω*. The normalized result is shown in Figure 1c. It can be seen that the top and bottom Ti layers make the main contribution to the total absorption, which is a characteristic of strong near‐field enhancement and energy dissipation at the metal dielectric interface.

As is widely known, when the effective impedance of the absorber matches the free space, the reflection will reach the minimum value, optical impendence matching plays an important role in areas of metamaterial absorbers. Absorption can be represented as [31]:

(2) $$A\left(\lambda \right)=1-R\left(\lambda \right)=1-{\left|\frac{Z-Z_{0}}{Z+Z_{0}}\right|}^{2}=1-\left|\frac{\sqrt{\mu }-\sqrt{\varepsilon }}{\sqrt{\mu }+\sqrt{\varepsilon }}\right|$$

where *Z*_0_ and *Z* are the effective impedance of free space and absorber, *µ* and *ε* are the effective permeability and permittivity of the absorber. This indicates that *ε* = *µ* is a key factor in achieving perfect absorption.

Therefore, to match the impedance with free space, permeability and permittivity are equally important. To explain the mechanism of broadband absorption, we calculated the equivalent impedance of the absorber by [32]:

(3) $$Z=\sqrt{\frac{{\left(1+S_{11}\right)}^{2}-S_{21}^{2}}{{\left(1-S_{11}\right)}^{2}-S_{21}^{2}}}$$

(4) $$Z=Z^{\prime }+iZ^{\prime \prime }$$

where *S*_11_ and *S*_21_ are the scattering parameters, the equivalent impendence is calculated from *S*_11_ and *S*_21_, and the impendence of the air is considered to be:

(5) $$Z_{\mathrm{air}}=1$$

the real and imaginary parts of the equivalent impedance both contribute to the reflectivity of the absorber, according to:

(6) $$R=\frac{{\left(Z^{\prime }-1\right)}^{2}+{\left(Z^{\prime \prime }\right)}^{2}}{{\left(Z^{\prime }+1\right)}^{2}+{\left(Z^{\prime \prime }\right)}^{2}}$$

perfect absorption occurs at the wavelength where the equivalent impendence is perfectly matched to the air, that is to say, the real part is equal to 1, and the imaginary part is equal to 0. This near perfect absorption is quantitatively explained by the impedance matching theory, where the impedance in Figure 1d approaches the ideal condition of free space (*Z*′ ≈ 1, *Z*” ≈ 0) precisely at the resonance wavelengths, minimizing reflection.

### 2.2 Unraveling the Absorption Mechanisms: Hybrid Plasmonic Resonances

**FIGURE 2:**
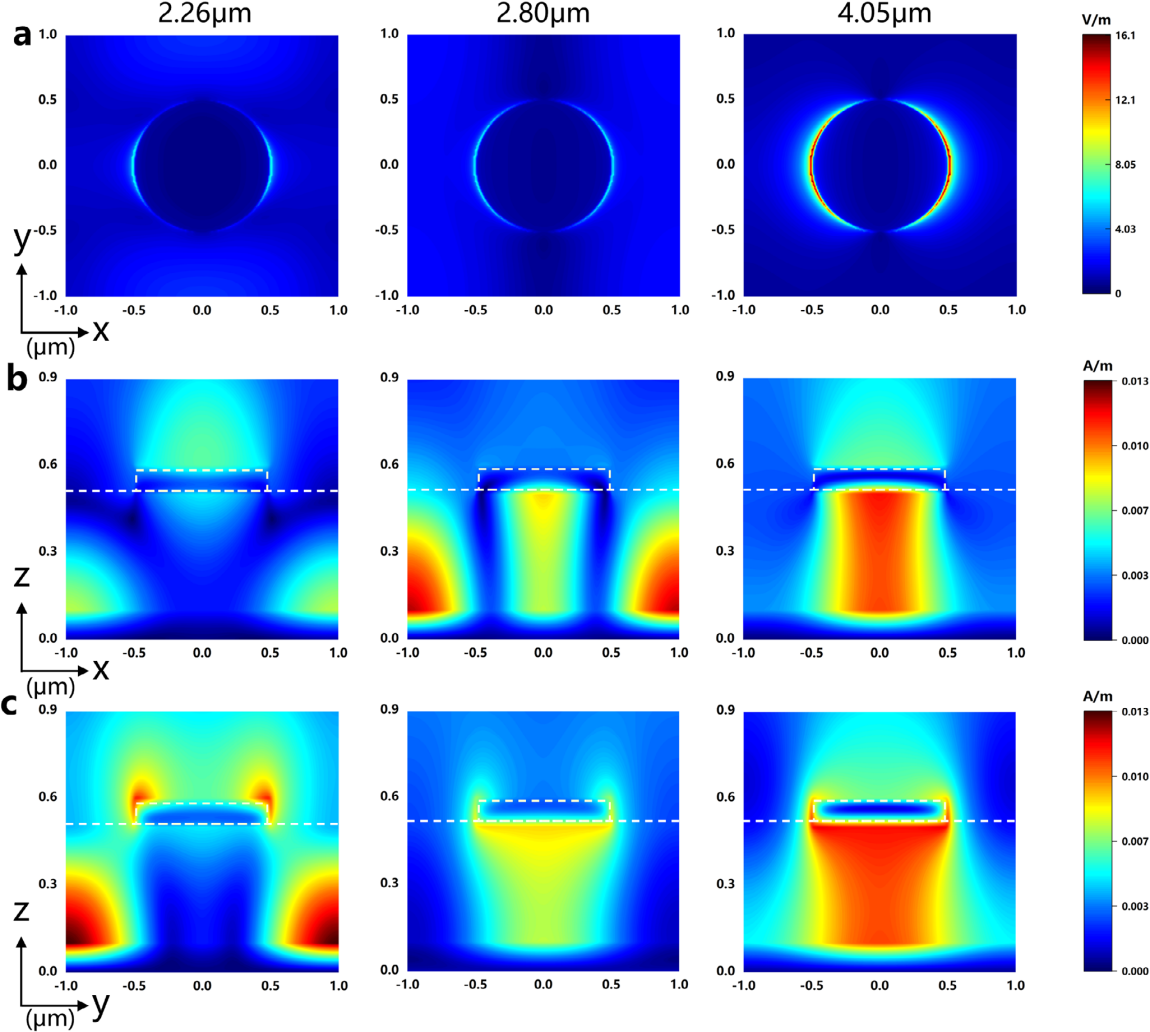
Electromagnetic field distributions of the proposed Ti/Al_2_O_3_/Ti absorber at its four resonant wavelengths in the MIR ranges. a) Electric field distributions in x‐y plane. And magnetic field distribution in b) x‐z plane (*y* = 0) and c) y‐z plane (*x* = 0).

The physical origin of the broadband absorption is unraveled by examining the electromagnetic field distributions at the resonance wavelengths, as depicted in Figure 2. The electric field profile on the x‐y plane (Figure 2a) shows strong localization within the gaps between adjacent unit cells, indicating the efficient excitation of surface plasmon polaritons. Correspondingly, the cross‐sectional magnetic field distributions in the x‐z and y‐z planes (Figure 2b,c) provide clear evidence of hybrid plasmonic resonances: at 2.26 µm, the magnetic field is concentrated beneath the gap between adjacent patches, characteristic of propagating surface plasmon resonance; at 2.80 µm, the field is strongly localized underneath the individual patch, signifying a dominant localized surface plasmon resonance; and at 4.05 µm, the field pattern suggests a mixed mode. This synergistic coupling of multiple resonance mechanisms is the cornerstone for achieving broadband performance with a simple, single‐sized meta‐atom design.

### 2.3 Loss Engineering with a Fe_3_O_4_ Interlayer for Enhanced Performance

**FIGURE 3:**
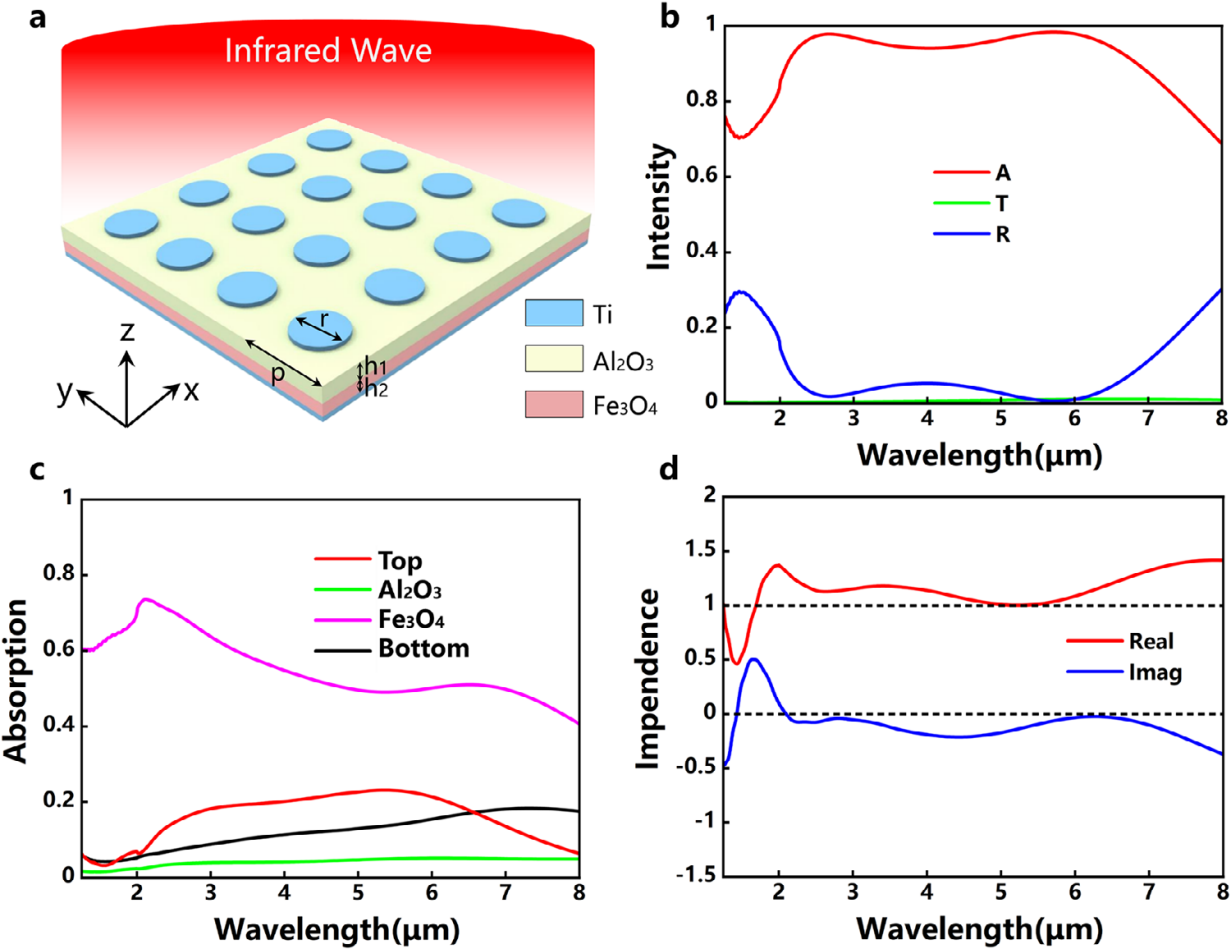
Characteristics of the Ti/Al_2_O_3_/Fe_3_O_4_/Ti four‐layers absorber. a) Structural diagram of the proposed Ti/Al_2_O_3_/Fe_3_O_4_/Ti absorber with the periodic circular top resonators. Specifically, *r* = 1 µm, *p* = 2 µm, *h*_1_ = 400 nm and *h*_2_ = 300 nm. The thickness of the top Ti layer is 30 nm, and the bottom Ti layer is 100 nm. b) Absorption, transmission and reflective spectrum of the proposed Ti/Al_2_O_3_/Fe_3_O_4_/Ti absorber. c) The contribution of each component of the proposed Ti/Al_2_O_3_/Fe_3_O_4_/Ti absorber to absorption. d) The equivalent impedance of the proposed Ti/Al_2_O_3_/Fe_3_O_4_/Ti absorber.

To transcend the performance limitations of the classic MIM structure for stringent stray‐light elimination applications, we introduced a lossy Fe_3_O_4_ interlayer, constructing a Ti/Al_2_O_3_/Fe_3_O_4_/Ti heterostructure. Figure 3 comprehensively characterizes this loss‐engineered absorber.​ The structural schematic in Figure 3a shows the incorporation of a 300‐nm‐thick Fe_3_O_4_ layer. The absorption spectrum in Figure 3b demonstrates a dramatic performance enhancement, achieving a remarkably smooth and ultra‐broadband absorption profile with an average absorptivity of 95.7% from 2.10 to 6.82 µm. This broadening effect is directly attributed to the loss engineering strategy, as confirmed by the power dissipation analysis in Figure 3c, which shows the Fe_3_O_4_ layer becomes the dominant contributor to absorption across the majority of the band. The consequent superior impedance matching over an extended wavelength range, illustrated in Figure 3d, further validates the effectiveness of our material‐level innovation in achieving a quasi‐ideal absorber response. The electromagnetic field distribution diagram of Ti/Al_2_O_3_/Fe_3_O_4_/Ti four‐layer structure absorbent is shown in Figure S1. Further explanation of the function of the Fe_3_O_4_ layer and the influence of its thickness on the overall absorption performance is shown in Figure S2.

### 2.4 Experimental Realization and Characterization on a Flexible Substrate

**FIGURE 4:**
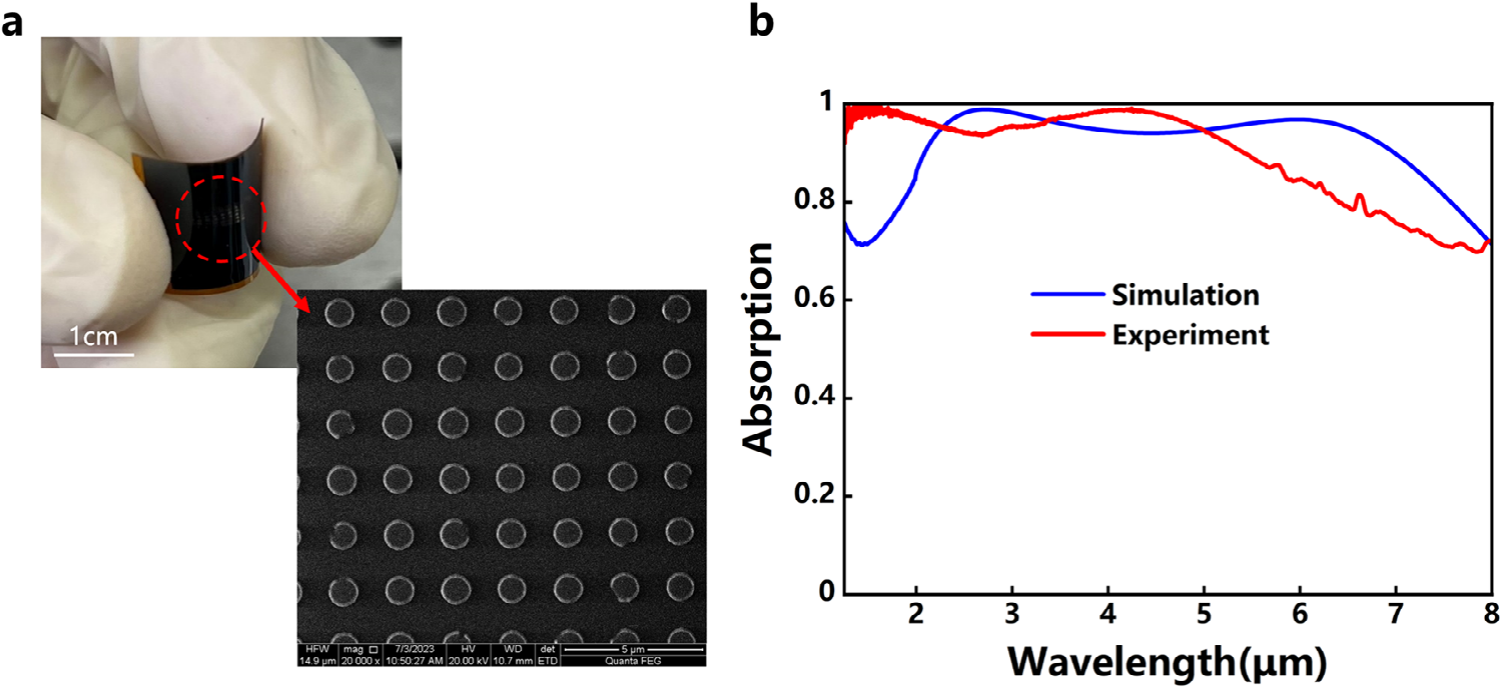
Experimental and simulated results of Ti/Al_2_O_3_/Fe_3_O_4_/Ti absorber, as well as performance integrated on flexible substrates. a) Image and SEM images of the proposed Ti/Al_2_O_3_/Fe_3_O_4_/Ti absorber fabricated on a flexible substrate. b) Experimental and simulated absorption spectrum of the proposed Ti/Al_2_O_3_/Fe_3_O_4_/Ti 4 layers absorbers integrated on flexible substrates.

The transition from simulation to experimental validation is a critical step for demonstrating the practical feasibility of our metamaterial design. We fabricated the optimized Ti/Al_2_O_3_/Fe_3_O_4_/Ti structure over large areas on flexible polyimide (PI) substrates using standard lithography and thin‐film deposition techniques. As shown in the optical and scanning electron microscopy (SEM) images in Figure 4a, the periodic array of the top Ti patterns was successfully created, confirming the structural integrity and scalability of our fabrication process. The presence of minor defects, which is inherent to large‐area nanofabrication, does not significantly compromise the overall optical performance, as the collective response of the metamaterial array dominates.

The experimental absorption spectrum, plotted alongside the simulation results in Figure 4b, exhibits excellent agreement, achieving an outstanding average absorption of 97.1% within the target 3–5 µm atmospheric window. The experimental results have a blue shift compared to the simulation results. For the difference between simulation and experimental results, the actual refractive index deviation of Ti, Al_2_O_3_, and Fe_3_O_4_ prepared may lead to partial deviation of resonance effect, and there may be some errors in the actual preparation size, such as inaccurate control of film thickness. Overall, these results validate our design strategy and emphasize the robustness of the manufacturing method for producing high‐performance, large‐area flexible metamaterial absorbers.

### 2.5 Robust Angular and Polarization Independence for Practical Use

**FIGURE 5:**
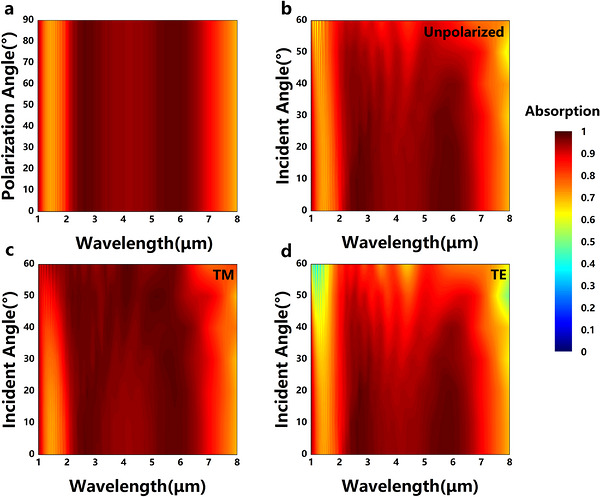
Dependence of the proposed Ti/Al_2_O_3_/Fe_3_O_4_/Ti absorber's performance. a) Absorption spectrum for incident polarization angles from 0–90°. And absorption spectrum for incident angles from 0–60° under b) Unpolarized light, c) TM–mode (x–polarized) and d) TE–mode (y–polarized).

For practical stray light elimination flexible devices, high absorption for unpolarized light within a wild incident angle range is an indispensable key characteristic. As shown in Figure 5a, when the polarization angle of the incident light gradually changes from 0° to 90°, the absorption spectrum does not change and shows the characteristic of polarization insensitivity. Moreover, it can be seen from Figure 5b that as the incident angle increases, both absorption peaks remain unchanged, but the absorption rate slightly decreases. Further, it is not difficult to see from Figure 5c that as the incident angle increases to 60°, the absorption peak dominated by PSPR remains unchanged, while the absorption peak dominated by LSPR undergoes a slight blue shift, but the absorption rate remains basically unchanged, indicates the absorber is insensitive to the incident angle under the TM mode. Moreover, it is shown in Figure 5d, that as the incident angle increases to 60°, the absorption peak dominated by PSPR still remains unchanged, while the absorption peak dominated by LSPR undergoes a slight red shift and the absorption rate decreases. However, the absorption rates also remain above 80%. Overall, the proposed absorber exhibits excellent characteristics of polarization and incident angle insensitivity, which has significant potential for practical applications.

### 2.6 Stray Light Suppression in a Practical Optical System

**FIGURE 6:**
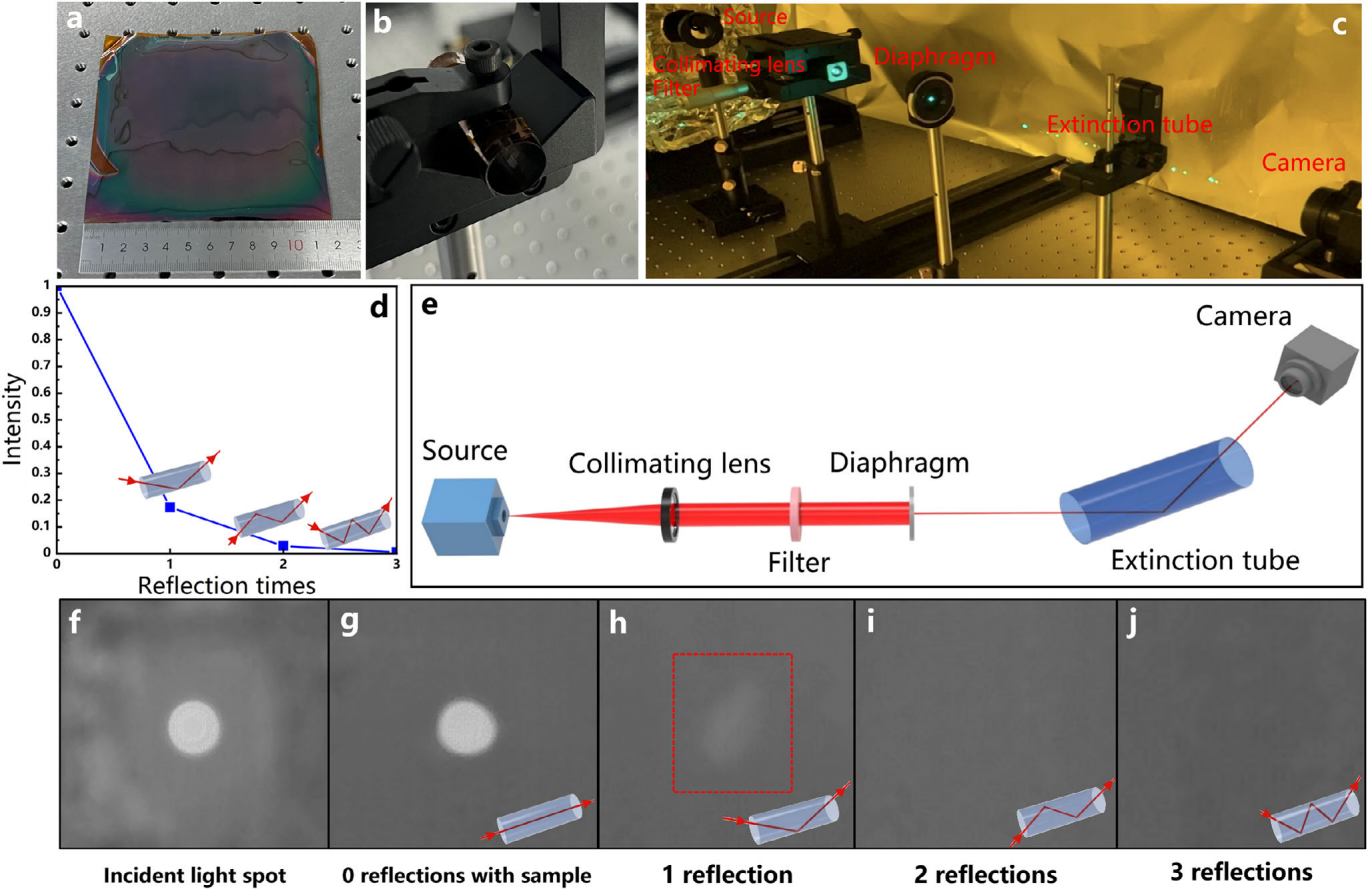
Absorption performance integrated on flexible substrate. a) Image of the large‐area extinction tube integrated on a flexible substrate and b) the extinction tube integrated on a flexible substrate is rolled into a cylindrical shape and fixed on a V‐shaped fixture. c) Image of the entire optical path. d) Light intensity after n reflections. e) Schematic diagram of optical path. And spot image with f) 0 reflections without extinction tube, g) 0 reflections with extinction tube, and after h) 1 reflection, i) 2 reflections and j) 3 reflections.

In terms of achieving large‐scale production, due to the long time required for large‐area preparation using laser direct writing and machine overheating issues, we used mask lithography technology to manufacture large‐area samples with unit structure diameters increased to 3 µm. Compared to the spectral lines of the 1 µm structure, its absorption spectrum has a redshift, but its average absorption rate in the 3–5 µm range remains at 84.4%. Therefore, the results can be directly compared with those in Figures 3 and 4. The specific experimental results are shown in Figure S3. The image of the large‐area sample integrated on a flexible substrate is shown in Figure 6a. Figure 6b shows the image of the extinction tube integrated on a flexible substrate, rolled into a cylindrical shape, and fixed on a V‐shaped groove fixture. Place the prepared cylinder in the optical path of the simulated optical system to eliminate stray light. The image of the entire optical path is shown in Figure 6c, and the entire optical path is collimated by laser. Figure 6e shows the schematic diagram of the optical path. Infrared light is emitted from the light source, collimated by a plano‐convex lens, through a filter, passed through a diaphragm, and finally passed through a cylindrical extinction tube to reach the infrared camera. Figure 6d shows the normalized light intensity after n reflections. It can be seen that after one reflection, the light intensity is only 17.4%, with an absorption rate of 82.6%. After the second and third reflections, the light intensity is 2.9% and 0.6%, respectively, with corresponding absorption rates of 97.1% and 99.4%, respectively. Figure 6f–j shows the spot images of 0 reflections without extinction tube, 0 reflections with extinction tube, and 1 to 3 reflections, respectively. It can be seen that compared to the spot where the extinction tube is placed, the stray light of the spot after placing the extinction tube is significantly reduced, improving the signal‐to‐noise ratio. The intensity of the light spot after the first reflection has significantly decreased, while the light spots from the second and third reflections are almost invisible, and the intensity of the third reflection is weaker compared to the second reflection.

### 2.7 Demonstration of Enhanced Imaging Contrast

**FIGURE 7:**
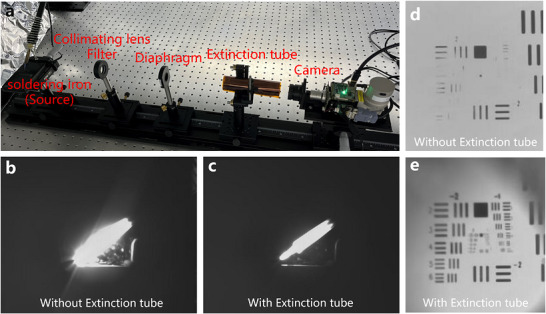
Integrated imaging performance on the camera. a) Integrated imaging optical path of extinction tube and infrared camera. And infrared image of soldering iron captured by b) traditional infrared camera and c) infrared camera integrated with the extinction tube. As well as infrared image of the USAF resolution test target captured by d) traditional infrared camera and e) infrared camera integrated with the extinction tube.

As shown in Figure 7a, the imaging optical path of the 673K soldering iron is captured by integrating the extinction tube with a traditional infrared camera. Figure 7b,c shows the infrared images of the 673K soldering iron obtained using a traditional infrared camera and an infrared camera integrated with the extinction tube, respectively. The absorber significantly improves the contrast of the image. If there is no absorber, the image contrast is too low to display the outline of the soldering iron. On the contrary, using a camera with an extinction tube reel allows for a clearer observation of the shape of the soldering iron. Similarly, as shown in Figure 7d,e, the identification of US Air Force resolution targets captured by traditional cameras is difficult due to the high intensity of target light and stray light. Infrared cameras integrated with extinction tubes can control the intensity of target light while reducing the interference of stray light, allowing the integrated camera to achieve a resolution of 0.63 lp mm^−1^. Overall, the device can achieve the function of eliminating stray light in the system while allowing target light to pass through, which realizes the application path of metamaterial absorbers in eliminating stray light in optical systems. Since the existing samples are processed through laser direct writing, there is still room for improvement in its extinction effect. In the future, it can be exposed and prepared under i‐line lithography machines or deep ultra lithography machines, which will result in smaller sizes, higher absorption rates, and higher extinction rates, while still allowing for large‐area preparation of flexible samples.

## 3 Discussion

In summary, we have successfully realized a large‐area, flexible metamaterial absorber through a loss engineered Ti/Al_2_O_3_/Fe_3_O_4_/Ti heterostructure that exhibits ultra‐broadband, high‐efficiency infrared extinction, with an average absorptivity of 97.1% in the 3–5 µm atmospheric window. This work not only elucidates the hybrid plasmonic resonance mechanisms underpinning the broadband absorption but also demonstrates, through the strategic incorporation of a lossy dielectric layer, a powerful strategy for achieving smooth and wideband optical performance. The successful fabrication of a flexible extinction tube and its integration into a practical optical system, resulting in a drastic reduction of stray light intensity to 0.6% and a substantial enhancement in imaging contrast, validates the immense application potential of this platform. This study paves the way for the development of next generation, miniaturized, and flexible optical devices, with promising implications for fields ranging from infrared imaging and stealth to electromagnetic shielding, and further performance enhancements can be anticipated through more advanced nanofabrication techniques.

## 4 Simulations Section

Theoretical simulations were conducted on the numerical absorption spectra and electromagnetic field distribution of absorption peaks of absorbers using the finite difference time domain method. The unit cell of the studied structure was simulated using periodic boundary conditions along the x and y axes and a perfectly matched layer propagating along the electromagnetic wave (z‐axis). Choose a grid precision of 8, set the computation time to 5000 fs, and auto‐shutoff the minimum value of 1 × 10^−5^. The refractive index of Ti comes from Rakic Lorentz‐Drude model's manual [33], while the refractive index of Fe_3_O_4_ comes from Querry's manual [34], and the refractive index of Al_2_O_3_ comes from Kischkat [35]. The plane wave is emitted from the top of the elemental surface, and the electromagnetic field distribution is illuminated by TM polarized plane waves. For oblique incidence, the incidence angle ranges from 0 ° to 60 °, with a step size of 10°.

## 5 Experimental Section

This study prepared Ti/Al_2_O_3_/Fe_3_O_4_/Ti structures. The substrate is PI, and for certain process steps such as photolithography, silicon with a crystal orientation of 100 (TIR< 3 µm, BOW< 10 µm, *R*a< 5 nm) is required as a support layer. For substrate cleaning, sonicate with NMP solution for 5 minutes, then sonicate with water for 5 minutes, and finally blow dry. Use evaporation coating equipment to coat a 100 nm Ti layer on the substrate. For the different dielectric layers of the proposed absorbent, 400 nm Al_2_O_3_ and 300 nm Fe_3_O_4_ layers were coated on the Ti layer. The vacuum degree of the coating is less than 5×10^−5 ^Pa, and the coating rate is 0.5 A/s. Then coat AZ5214 ultraviolet positive photoresist on the substrate. MLA maskless laser direct writing Lithography is used to define the pattern of the top Ti layer. To clean the strip, immerse the structure completely in NMP solution and heat it to 80°C. After 2 min, the Ti that needs to be peeled off clearly drips into the solution. Then use a new solution, soak the structure for another 20 min, and rinse with clean water. Finally, the proposed structure is obtained.

The infrared Fourier transform spectrometer measurement system is used to demonstrate the reflection spectra of Ti/Al_2_O_3_/Fe_3_O_4_/Ti absorbers. To determine the absolute reflectance *R*, the measured reflectance is completely canceled out by the opaque metal mirror in the transmission channel of the reference Au mirror, and is directly determined as *A* = 1‐*R* based on the absorption of *A* = 1‐*R*‐*T*. The measurement range is 600–7500 cm^−1^. Silicon nitrogen rods are used as infrared light sources and thermal module infrared industrial camera is used to capture the infrared light spots.

## Conflicts of Interest

The authors declare no conflicts of interest.

## Supporting information




**Supporting File**: advs75513‐sup‐0001‐SuppMat.docx.

## Data Availability

The data that support the findings of this study are available from the corresponding author upon reasonable request.

## References

[1] S. K. He , Y. Tian , H. M. Zhou , et al., “Review for Micro‐Nano Processing Technology of Microstructures and Metadevices,” Advanced Functional Materials 35 (2025): 2420369.

[2] W. J. Liu , Y. F. Wu , X. Z. D. Bao , L. Sun , Y. E. Xie , and Y. P. Chen , “High‐Performance Infrared Self‐Powered Photodetector Based on 2D Van der Waals Heterostructures,” Advanced Functional Materials 35 (2025): 2421525.

[3] K. Mahato , T. Saha , S. C. Ding , S. S. Sandhu , A. Y. Chang , and J. S. Wang , “Hybrid Multimodal Wearable Sensors for Comprehensive Health Monitoring,” Nature Electronics 7 (2024): 735–750.

[4] Z. He , Y. Zhang , X. Tong , L. Li , and L. V. Wang , “Quantum Microscopy of Cells at the Heisenberg Limit,” Nature Communications 14 (2023): 2441. 10.1038/s41467-023-38191-4 PMC10147633 37117176

[5] D. E. Tranca , S. G. Stanciu , R. Hristu , et al., “Diffraction‐Induced Artifacts in Scattering‐Type Scanning Near‐Field Optical Microscopy due to Lateral and Longitudinal Inhomogeneities,” Optics & Laser Technology 192 (2025): 113848.

[6] Y. He , C. Zhang , B. Zhang , and Z. Chen , “FSPnP: Plug‐and‐Play Frequency–Spatial‐Domain Hybrid Denoiser for Thermal Infrared Image,” IEEE Transactions on Geoscience and Remote Sensing 62 (2024): 5000416.

[7] N. Zhang , L. Wu , W. Gao , Q. Zhao , N. Huo , and J. Li , “Near‐Infrared, Self‐Powered and Polarization‐Sensitive Photodetector Based on GeSe–MoTe_2_ p–n Heterojunction,” Advanced Materials Interfaces 9 (2022): 2200150.

[8] B. Ma , Q. Chen , H. Wang , Y. Xue , Z. Ma , and J. Liu , “Stray Light Measurement for Space Optical Systems Using the Time‐Domain Method ,” Optics Express 33 (2025): 554196. 10.1364/OE.554196 40798646

[9] C. M. Tsai , S. Vyas , and Y. Luo , “Common‐Path Digital Holographic Microscopy Based on a Volume Holographic Grating for Quantitative Phase Imaging,” Optics Express 32 (2024): 514225. 10.1364/OE.514225 38439461

[10] C. Shen , S. Xu , Z. Chen , N. Ji , J. Yang , and J. Zhang , “Fluorobenzene and Water‐Promoted Rapid Growth of Vertical Graphene Arrays by Electric‐Field‐Assisted PECVD,” Small 19 (2023): 2207745. 10.1002/smll.202207745 36650988

[11] T. K. Truong , G. T. Yuk , J. B. Kim , H. Youn , and J. Rho , “Super Black Coating on the Commercial Black Anodized Al(6061) by Direct and Scalable CVD–Growth of Carbon Nanofibers,” Advanced Materials Interfaces 11 (2024): 2400032.

[12] X. Zhang , H. Kang , X. Jia , et al., “Construction of Broad‐Spectrum Light Absorption Inorganic Ultra‐Black Coating with Multi‐Scale Light‐Absorbing Structures Based on Hollow CuCr_2_O_4_ Spheres,” Materials Today Physics 58 (2025): 101884.

[13] Y. Di , K. Ba , X. Wang , et al., “Advanced Architectures and Emerging Materials for High‐Operating‐Temperature Infrared Photodiodes,” Advanced Materials 37 (2025): 08115. 10.1002/adma.202508115 PMC13549255 40922390

[14] H. Jiao , X. Wang , Y. Chen , et al., “HgCdTe/Black Phosphorus van der Waals Heterojunction for High‐Performance Polarization‐Sensitive Midwave Infrared Photodetector,” Science Advances 8 (2022): abn1811. 10.1126/sciadv.abn1811 PMC9094662 35544556

[15] P. Wu , L. Ye , L. Tong , et al., “Van der Waals Two‐Color Infrared Photodetector,” Light: Science & Applications 11 (2022): 6. 10.1038/s41377-021-00694-4 PMC8720310 34974520

[16] S. Zhang , S. An , M. Dai , et al., “Chalcogenide Metasurfaces Enabling Ultra‐Wideband Detectors from Visible to Mid‐Infrared,” Advanced Science 12 (2025): 2413858. 39968970 10.1002/advs.202413858 PMC11984864

[17] S. Zhang , L. Han , K. Xiao , et al., “H‐BN‐Encapsulated Uncooled Infrared Photodetectors Based on Tantalum Nickel Selenide,” Advanced Functional Materials 33 (2023): 2305380.

[18] D. Wu , Z. Mo , X. Li , et al., “Integrated Mid‐Infrared Sensing and Ultrashort Lasers Based on Wafer‐Level Td‐WTe_2_ Weyl Semimetal,” Applied Physics Reviews 11 (2024): 041401.

[19] Y. Miao , Y. Y. Nie , H. J. Zou , et al., “Carbon Dots via Synergistic Surface‐Oxidation and Rigidity Enhancement with Ultra‐Narrow Emission for Sensitive Near‐Infrared Imaging and Sepsis Therapy,” Advanced Functional Materials 36 (2025): 2513584.

[20] J. Y. Qi , X. Q. Liu , Z. J. Liu , et al., “Ultrawide Spectrum Metallic Plane Blackbody with Extremely High Absorption from 0.2 to 25 µm,” Advanced Science 12 (2025): 2411448. 39569754 10.1002/advs.202411448 PMC11727123

[21] Z. Ren , Z. Yang , W. Mu , T. Liu , X. Liu , and Q. Wang , “Ultra‐Broadband Perfect Absorbers Based on Biomimetic Metamaterials with Dual Coupling Gradient Resonators,” Advanced Materials 37 (2025): 2416314. 10.1002/adma.202416314 39703098

[22] B. X. Wang , X. Qin , G. Duan , G. Yang , W. Q. Huang , and Z. Huang , “Dielectric‐Based Metamaterials for Near‐Perfect Light Absorption,” Advanced Functional Materials 34 (2024): 2402068.

[23] B. X. Wang , C. Xu , G. Duan , W. Xu , and F. Pi , “Review of Broadband Metamaterial Absorbers: from Principles, Design Strategies, and Tunable Properties to Functional Applications,” Advanced Functional Materials 33 (2023): 2213818.

[24] W. Hu , R. Yan , Y. Ma , et al., “Vertical Pyro‐Phototronic Effect and Lateral Photothermoelectric Effect in Perovskite Single Crystals‐Based Photodetector for Narrowband and Broadband Dual‐Modal Optical Communications,” Laser & Photonics Reviews 19 (2025): 2401990.

[25] C. Li , G. Wang , M. Peng , et al., “Reconfigurable Origami/Kirigami Metamaterial Absorbers Developed by Fast Inverse Design and Low‐Concentration MXene Inks,” ACS Applied Materials & Interfaces 16 (2024): 4c07084. 10.1021/acsami.4c07084 39078617

[26] K. Li , Y. Liang , Y. Liu , and Y. S. Lin , “A Microfluidics Platform for Simultaneous Evaluation of Sensitivity and Side Effects of Anti‐Cancer Drugs Using a Three‐Dimensional Culture Method,” Microsystems & Nanoengineering 11 (2025): 2. 39747285 10.1038/s41598-024-84297-0 PMC11697305

[27] Y. Ma , H. Zhao , N. Luo , F. Chen , and Q. Fu , “From Magnetoelectric Core–Shell Structure to Compound Eye‐Inspired Metamaterials: Multiscale Design of Ultra‐Wideband Electromagnetic Wave Absorber Device,” Small 21 (2025): 2502186. 10.1002/smll.202502186 40275826

[28] N. Qu , G. Xu , Y. Liu , et al., “Multi‐Scale Design of Metal–Organic Framework Metamaterials for Broad‐Band Microwave Absorption,” Advanced Functional Materials 35 (2025): 2402923.

[29] J. Shi , J. Luo , C. Liu , et al., “From Directional to Omnidirectional: Meta‐Devices for Ultrabroadband Sound Absorption with Near‐Causality‐Limit Performance,” Materials Horizons 12 (2025): 9262–9271. 40758364 10.1039/d5mh00404g

[30] W. Li and J. Valentine , “Metamaterial Perfect Absorber Based Hot Electron Photodetection,” Nano Letters 14 (2014): 3510–3514. 24837991 10.1021/nl501090w

[31] Y. Zhou , Z. Qin , Z. Liang , et al., “Ultra‐Broadband Metamaterial Absorbers from Long to Very Long Infrared Regime,” Light: Science & Applications 10 (2021): 138. 10.1038/s41377-021-00577-8 PMC8257711 34226489

[32] A. Tittl , A. K. U. Michel , M. Schaeferling , et al., “A Switchable Mid‐Infrared Plasmonic Perfect Absorber with Multispectral Thermal Imaging Capability,” Advanced Materials 27 (2015): 1502023. 10.1002/adma.201502023 26173394

[33] K. Tang , K. Dong , J. Li , et al., “Temperature‐Adaptive R adiative Coating for All‐Season Household Thermal Regulation,” Science 374 (2021): abf7136. 10.1126/science.abf7136 34914515

[34] A. Thomas , L. Lethuillier‐Karl , K. Nagarajan , et al., “Tilting a Ground‐State Reactivity Landscape by Vibrational Strong Coupling,” Science 363 (2019): 615–619. 30733414 10.1126/science.aau7742

[35] O. Reshef , I. De Leon , M. Z. Alam , and R. W. Boyd , “Nonlinear Optical Effects in Epsilon‐Near‐Zero Media,” Nature Reviews Materials 4 (2019): 535–551.

