# Supplementary material for: A Flexible Metamaterial Absorber via Loss Engineering for Large‐Area Ultra‐Broadband Infrared Extinction
Source: Adv Sci (Weinh). 2026 May 7;13(42):e75513. doi: 10.1002/advs.75513 (PMC13335472; doi:10.1002/advs.75513)
Supplement: Supplementary file 1 — Supporting File: advs75513‐sup‐0001‐SuppMat.docx. [file ADVS-13-e75513-s001.docx]

**Supplementary information**

**A Flexible Metamaterial Absorber via Loss Engineering for Large-Area Ultra-Broadband Infrared Extinction**

Zhe Wu^1^，Zhongzhu Liang^*,1^，Xiaoyan Shi^1^，Fuming Yang^1^，Enzhu Hou^1^，Jihui Jiang^1^，Xintong Wei^1^，Siyu Guo^1^，Bing Sun^1^，Qingxin Tang^1^，David R. Smith^2^，Haiyang Xu^*,1^

^1^State Key Laboratory of Integrated Optoelectronics and Key Laboratory of ultraviolet Light-Emitting Materials and Technology of Ministry of Education, College of Physics, Northeast Normal University, Changchun 130024, China

^2^Center for Metamaterials and Integrated Plasmonics, Department of Electrical and Computer Engineering, Duke University, Durham, NC 27708, USA

* e-mail: [liangzz@nenu.edu.cn](mailto:liangzz@nenu.edu.cn), hyxu@nenu.edu.cn

Section S1: Electromagnetic Field Analysis of the proposed Ti/Al_2_O_3_/Fe_3_O_4_/Ti Absorber.


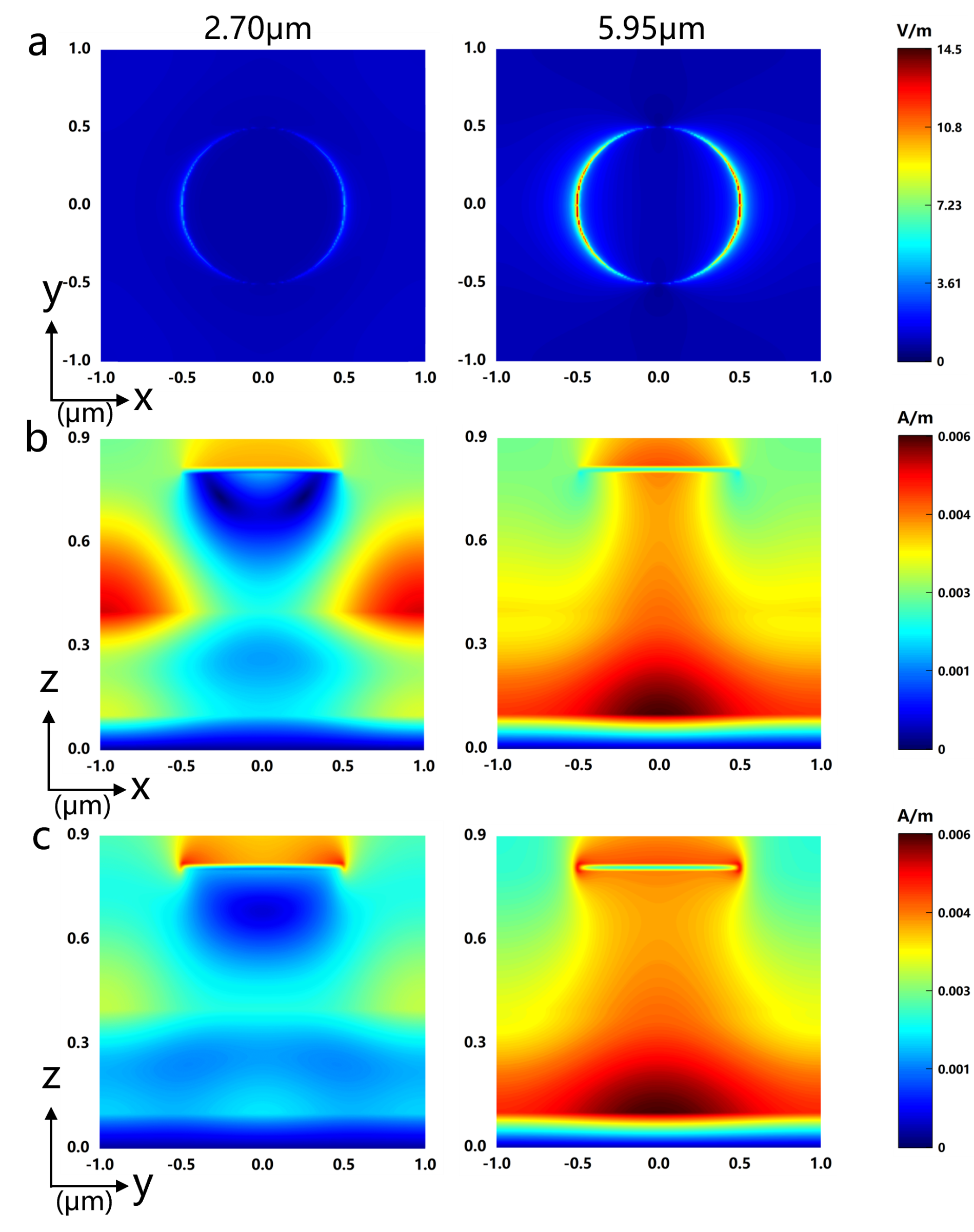


**Fig. S1 Electromagnetic field distributions of the proposed Ti/Al_2_O_3_/Fe_3_O_4_/Ti absorber at its three resonant wavelengths in the MIR ranges.** a) Electric field distributions in the top layer (x-y plane). b) Magnetic field distribution in the dielectric (x-z plane, y=0). c) Magnetic field distribution in the dielectric (y-z plane, x=0).

The electromagnetic field distribution of the Ti/Al_2_O_3_/Fe_3_O_4_/Ti four-layer structure is shown in **Fig.S1**a-c. Compared with the electromagnetic field of the Ti/Al_2_O_3_/Ti three-layer structure absorber, it can be seen that the electric field of the Ti/Al_2_O_3_/Fe_3_O_4_/Ti four-layer structure is still distributed on both sides of the surface pattern. Therefore, light is coupled into the air gap between adjacent crystal cells, which also generates SPP induced light absorption. As shown in Figures S1b, c, the magnetic field at 2.70μm is also mainly concentrated in the dielectric layer below the two adjacent surface patterns. However, the difference is that the magnetic field is mainly localized at the interface of the dielectric layers Fe_3_O_4_ and Al_2_O_3_, still the PSPR is mainly excited. Furthermore, the magnetic field at 5.95μm is still mainly concentrated at the interface between the dielectric layer Al_2_O_3_ below the surface pattern and the bottom Ti layer, indicating that PSPR dominates the absorption of the peak at 5.95μm. Therefore, the broadband absorption of the Ti/Al_2_O_3_/Fe_3_O_4_/Ti four-layer structure absorber is caused by the hybrid mode of SPP and PSPR and LSPR localized in different layers.

Section S2: Comparison of absorption performance between Fe_3_O_4_ layers and multilayer structures, and the influence of Fe_3_O_4_ layer thickness on overall absorption performance.


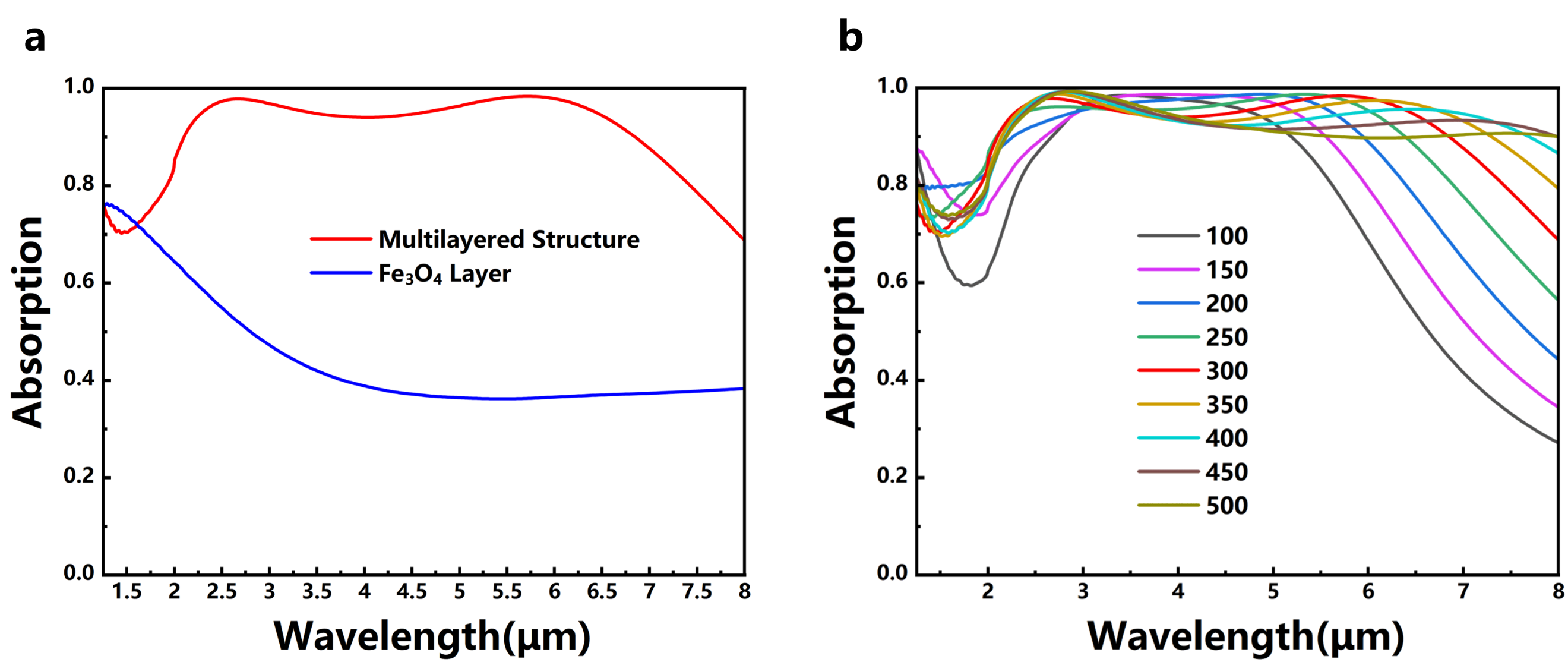


**Fig. S2 The function and parameters of Fe_3_O_4_ layer and its influence on the overall absorption performance.** a) Comparison of absorption performance between Fe_3_O_4_ layers and multilayer structures. b) Influence of Fe_3_O_4_ layer thickness from 100 to 500nm on overall absorption performance.

In order to further illustrate the function of Fe_3_O_4_ layer, we made a theoretical model of Fe_3_O_4_ layer as well as the multi-layer structure for comparison of their absorption performance, as shown in Figure S2a, to further demonstrate the function of Fe_3_O_4_ layer. The average absorption rate of Fe_3_O_4_ layer at 3-5μm is 40.7%, due to its inherent absorption properties, the absorption rate of the multi-layer structure compatible with Fe_3_O_4_ layer is increased and its absorption bandwidth is widened. The influence of the thickness of Fe_3_O_4_ layer on the overall absorption performance is shown in Figure S2b. As the thickness increases from 100nm to 500nm, the bandwidth of the overall absorption peak gradually widens, but its absorption rate in the long wave band gradually decreases. In addition, when the thickness of Fe_3_O_4_ layer is less than 200nm, the overall absorption peak at short wavelengths will decrease. Overall, the addition of Fe_3_O_4_ loss layer is crucial for the overall device performance.

Section S3: Absorption performance of a flexible roll shaped absorber with a size of 3μm of the proposed Ti/Al_2_O_3_/Fe_3_O_4_/Ti Absorber.


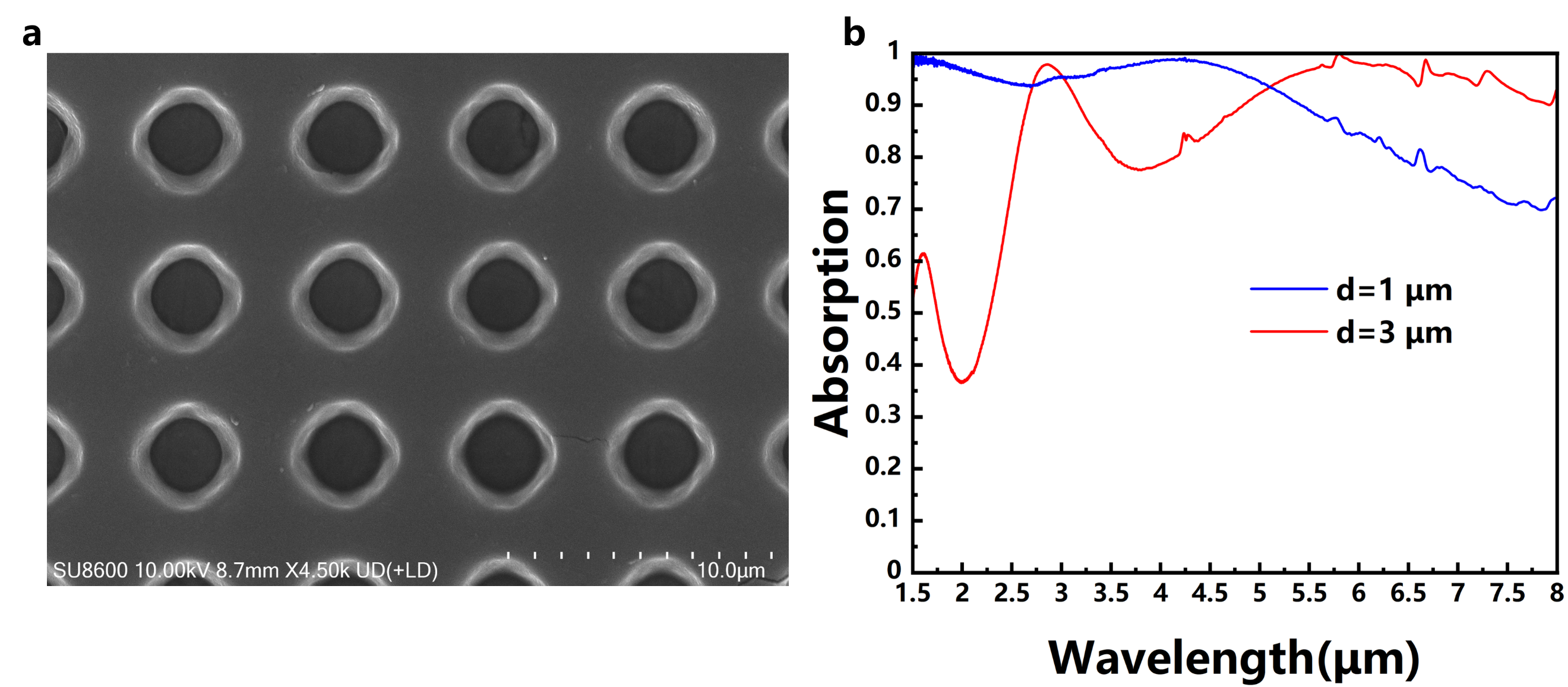


**Fig. S3 Experimental results of integrating Ti/Al_2_O_3_/Fe_3_O_4_/Ti absorbers with a diameter of 3μm on flexible substrates.** a) SEM images of the proposed Ti/Al_2_O_3_/Fe_3_O_4_/Ti 4 layers absorbers with a diameter of 3μm. b) Comparison of experimental absorption spectrum of the proposed Ti/Al_2_O_3_/Fe_3_O_4_/Ti 4 lays absorbers with sizes of 1μm and 3μm.

**Figure S3**a shows the SEM image of the proposed Ti/Al_2_O_3_/Fe_3_O_4_/Ti four-layer absorber with a diameter of 3μm. Due to the fact that the surface pattern actually prepared has a narrow upper and wide lower structure, there will be a white edge around the pattern. The experimental spectral comparison between the 1μm and 3μm sizes of the 4-layer absorber is shown in Figure S3b. Compared to the device with a size of 1μm, the spectrum of the device with a size of 3μm has a red shift. Two absorption peaks can be observed in the MIR band, located at 2.9μm and 6.0μm, with absorption rates of 97.8% and 99.6%, respectively. The average absorption rate between 3-5μm is 84.4%. Overall, compared to the structure with a size of 1μm, the experimental results of the structure with a size of 3μm showed a redshift, but the spectral curve still maintained the same trend as the simulation results. In addition, an absorption peak at a wavelength less than 1.5μm was also displayed due to a certain degree of redshift during simulation.

Section S4: Investigation of scattering parts and performance of inhibitory effects on different temperature targets.


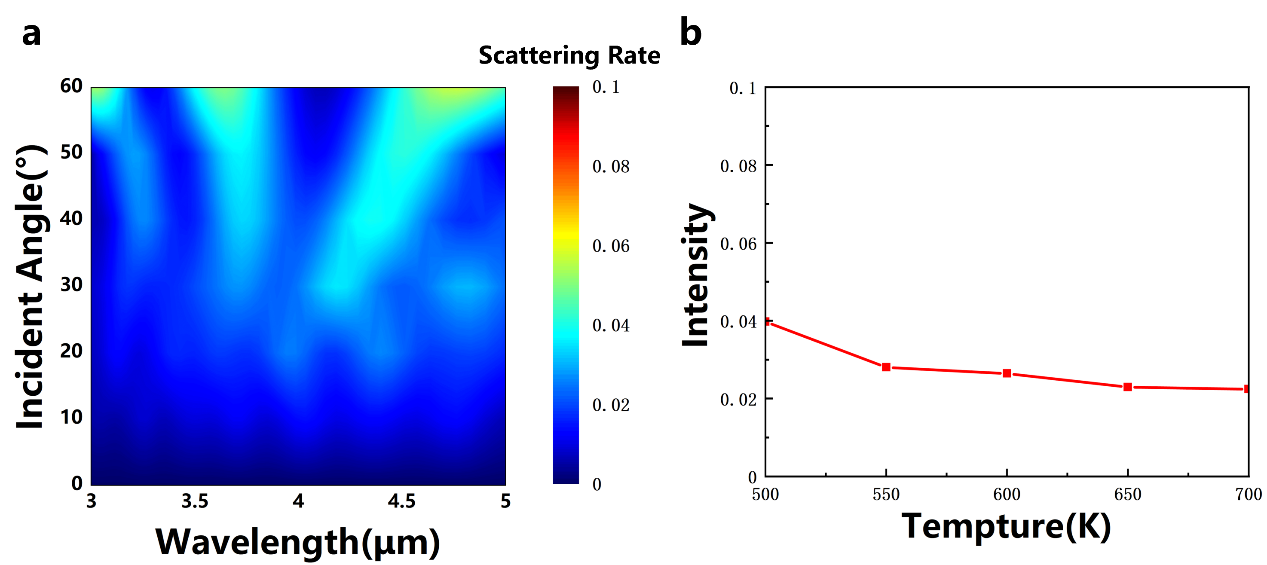


**Fig. S4 Scattering part and suppression performance of different temperature targets.** a) Scattering spectrum for incident angles from 0–60°. b) Suppression performance of targets at different temperatures.

To further investigate the scattering parts of incident light, such as diffuse scattering and non-specular reflection, we analyzed the relationship between the scattering rate of light and the incident angle, as shown in Figure **S4**a. It can be seen that as the incident angle increases from 0 to 60°, the scattering rate of light slightly increases from 0%, but the overall scattering rate does not exceed 6%. Overall, scattering has little effect on absorption, and the proposed device has good absorption performance. Furthermore, we also discussed the suppression performance of the target at different temperatures using samples with a size of 1μm. As shown in Figure S4b, it can be seen that as the target temperature increases from 500K to 700K, the reflected light intensity gradually decreases, indicating that its suppression performance on the target slightly improves with the increase of temperature in this temperature range.

Section S5: The influence of bending frequency on the absorption performance of the proposed Ti/Al_2_O_3_/Fe_3_O_4_/Ti absorber.


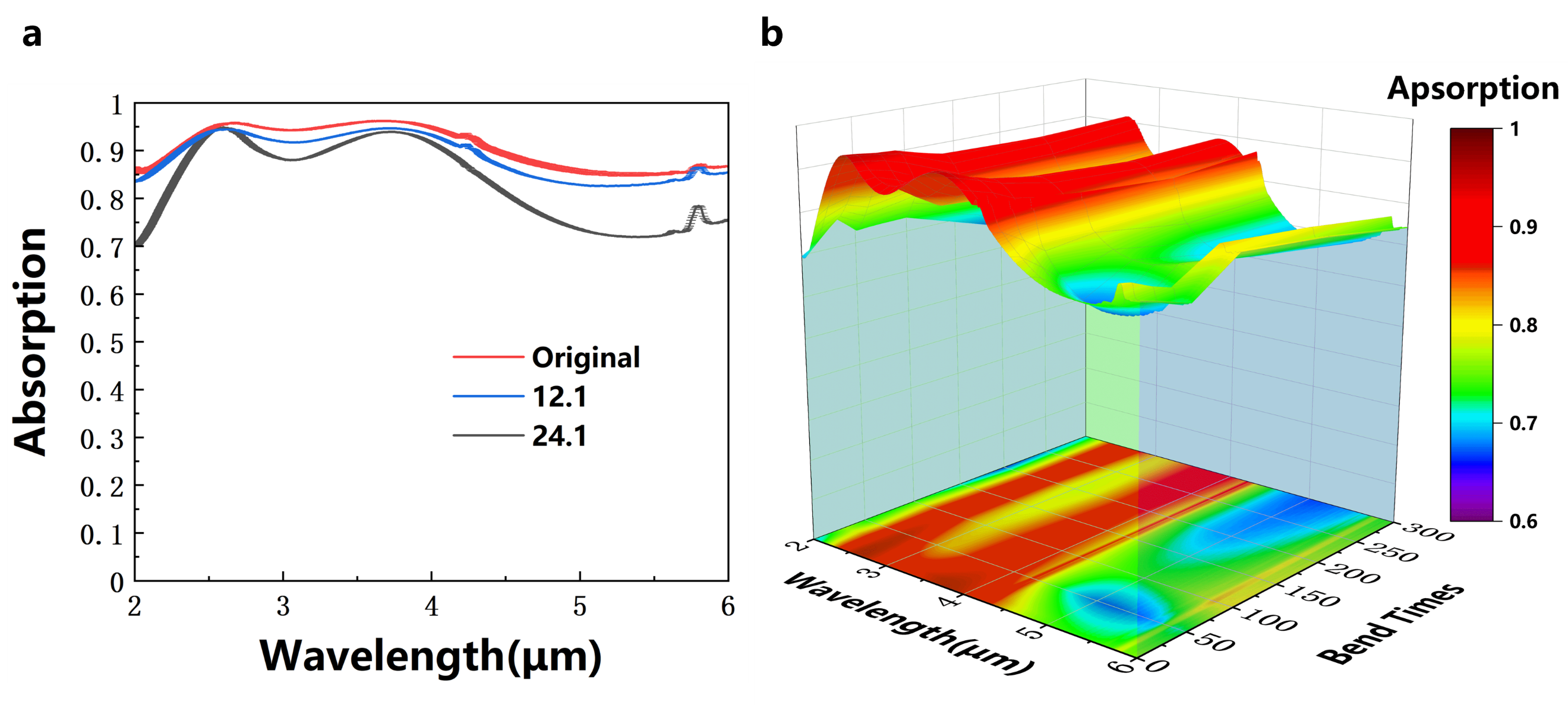


**Fig. S5 Experimental results of bending performance of Ti/Al_2_O_3_/Fe_3_O_4_/Ti absorber.** a) under different curvatures b) different bending times

**Figure S5**a shows the relationship between the absorption performance and curvature of the designed flexible extinction film. It can be observed that as the curvature increases, the absorption performance of the extinction film slightly decreases. However, at a curvature of 24.1, an average absorption rate of over 85% can still be achieved in the 3-5μm wavelength range, demonstrating good bending stability. Similarly, Figure S5b illustrates the relationship between the absorption performance and the bending and the number of bending cycles of the designed flexible matting film. It can be observed that as the bending frequency increases, the absorption performance of the matting film slightly decreases. However, after 300 bends, the overall absorption rate still exceeds 90%, with a decrease of less than 10%. This demonstrates the good bending resistance and practicality of the flexible matting film, further highlighting its potential in practical applications.

Section S6: Comparison with recent representative works incorporated with lossy layers.

**Table S1.** Comparison of representative works with lossy layers.

| Refs. | Operation Bandwidth | Total Absorption |
| --- | --- | --- |
| Chirumamilla et al. ^[1]^ | 2μm (0.3-2.3μm) | 94% |
| Ma et al. ^[2]^ | 0.4μm (0.4-0.8μm) | 96.88% |
| Zhou et al. ^[3]^ | 6μm (8-14μm)  16μm (14-30μm) | 95%  92% |
| Ding et al. ^[4]^ | 0.925μm (0.9-1.825μm) | >90% |
| Khosravi et al. ^[5]^ | 1.9μm (0.4-2.3μm) | 95.25% |
| Our work | 2μm (3-5μm) | 97.1% |

References

[1] M. Chirumamilla, A. Chirumamilla, Y. Yang, A. S. Roberts, P. K. Kristensen, K. Chaudhuri, A. Boltasseva, D. S. Sutherland, S. I. Bozhevolnyi, K. Pedersen, *Advanced Optical Materials.* **2017**, 5, 1700552.

[2] Y. Ma, J. Hu, W. Li, Z. Yang, *Nanomaterials.* **2023**, 13, 2726.

[3] Y. Zhou, Z. Qin, Z. Liang, D. Meng, H. Xu, D. R. Smith, Y. Liu, *Light-Science & Applications.* **2021**, 10, 138.

[4] F. Ding, J. Dai, Y. Chen, J. Zhu, Y. Jin, S. I. Bozhevolnyi, *Scientific Reports.* **2016**, 6, 39445.

[5] R. Khosravi, Y. E. Monfared, M. Qasymeh, *Results in Physics.* **2022**, 36, 105470.
